# Flexural Properties and Fracture Behavior of CF/PEEK in Orthogonal Building Orientation by FDM: Microstructure and Mechanism

**DOI:** 10.3390/polym11040656

**Published:** 2019-04-10

**Authors:** Qiushi Li, Wei Zhao, Yongxiang Li, Weiwei Yang, Gong Wang

**Affiliations:** 1 CAS Key Laboratory of Space Manufacturing Technology, Technology and Engineering Center for Space Utilization, Chinese Academy of Sciences, Beijing 100094, China; liqiushi17@csu.ac.cn (Q.L.); liyongxiang@csu.ac.cn (Y.L.); yangweiwei0811@163.com (W.Y.); 2 University of Chinese Academy of Science, Beijing 100049, China

**Keywords:** 3D printing, carbon fiber, PEEK, flexural property, crystallization, fracture mode

## Abstract

Fused deposition modeling possesses great advantages in fabricating high performance composites with controllable structural designs. As such, it has attracted attention from medical, automatic, and aerospace fields. In this paper, the influence of short carbon fibers (SCFs) and the orthogonal building orientation on the flexural properties of printed polyether ether ketone (PEEK) composites are systematically studied. The results show that the addition of SCFs raises the uniform nucleation process of PEEK during 3D printing, decreases the layer-to-layer bonding strength, and greatly changes the fracture mode. The flexural strength of vertically printed PEEK and its CF-reinforced composites show strengths that are as high as molded composites. X-ray micro-computed tomography reveals the microstructure of the printed composites and the transformation of pores during bending tests, which provides evidence for the good mechanical properties of the vertically printed composites. The effect of multi-scale factors on the mechanical properties of the composites, such as crystallization in different positions, layer-by-layer bonding, and porosity, provide a successful interpretation of their fracture modes. This work provides a promising and cost-effective method to fabricate 3D printed composites with tailored, orientation-dependent properties.

## 1. Introduction

Three-dimensional printing technology provides a promising technique for small-batch fabrication of highly customized objects that have a required performance that exceeds far beyond those of conventional manufacturing methods. Such technology has enabled the creation of composites that possess widespread applications in medical, automotive, and aerospace fields [1,2,3,4,5]. Specifically, polyether ether ketone (PEEK) is a versatile, high-performance material that can be fabricated by fused deposition modeling (FDM) due to its excellent heat resistance and strength-to-weight ratio [6,7,8].

However, an issue that currently limits its use in printed parts is its poor layer-to-layer bonding, non-uniform crystallization behavior, and residual thermal stress, which results from the inherent printing process. For printed PEEK parts, the layer-to-layer bonding in the *Z*-direction has been determined to be much lower than the corresponding in-plane strength, especially for particle-reinforced composites [9,10]. Moreover, the rapid crystallization behavior of printed PEEK parts also causes thermal stresses and unstable mechanical properties, resulting in the high probability of printing process failures. Several possible approaches, such as the addition of reinforced materials [11,12], optimizing printing parameters [13,14], and preheating [15,16], have been studied to improve the bonding strength of PEEK, and the obtained parts, with high mechanical performance. For example, Yan successfully fabricated high-performance carbon fiber (CF)/PEEK composites by selective laser sintering and investigated the influence of the processing parameters on their rheological properties [17]. Berretta and Stepashkin investigated the influence of carbon nanotubes and carbon fibers (CFs) on printed PEEK and found that more pores exist in printed PEEK composites [18,19]. Yang found that the crystallinity and mechanical properties of PEEK composites are highly dependent on the printing parameters and thermal treatment [20]. Obviously, studies on PEEK have revealed that the mechanical properties of printed parts are inferior to the properties of their injection molding parts due to their poor bonding strength and the presence of internal pores. Moreover, the effect of the addition of reinforcements on the crystallinity and fracture mode of printed PEEK matrix composites are not clear.

The influence of reinforced materials in the FDM process has attracted widespread attention. Carbon fiber is one of the typical reinforced materials used in the FDM process, which could enhance the mechanical properties of printed parts, such as in PLA, ABS, and PA12 et al. [21,22,23,24]. Ferreira et al. [21] studied the effect of deposition direction and carbon fiber on tensile strength of printed PLA and CF/PLA composites. Ning et al. [22] discovered that addition of CF into ABS matrix could increase tensile strength and Young’s modulus, but decrease toughness, yield strength, and ductility of printed parts. Liao et al. [23] reported that the addition of 10 wt % CF to the PA12 matrix could obviously increase the mechanical properties and thermal conductivity along the printing direction without reducing impact strength. Above all, the highly oriented CF along with deposition direction was believed for the increased strength. Meanwhile, the presence of CF in printed composites also increased the voids and decreased the layer-to-layer bonding strength to some extent. Up to now, it seems that there are contradictions between the increased mechanical property and more porosity. The role of inherent porosity in mechanical properties and fracture mode of printed CF reinforced composites still need to be determined.

To date, there has been only limited research regarding the incorporation of reinforced materials in the FDM process that could simultaneously overcome the low layer-to-layer bonding strength and adjust the crystallization behavior of printed PEEK parts. As such, this study focuses on the following: (1) to develop a combined FDM strategy that is based on the addition of CF into PEEK (CF/PEEK) and PEEK that is printed in the orthogonal building orientation; and (2) to investigate the influence of CF on PEEK’s microstructure and the flexural properties of the printed parts. In this work, the orthogonal PEEK and CF/PEEK printed composites were prepared by FDM, and then compared with injection-molding specimens. The results show that the vertically printed composites possess high flexural performance that are commensurate with those of injection-molding specimens. Their crystallinity behavior and microstructure are carefully investigated by differential scanning calorimetry (DSC) and X-ray micro-computed tomography (X-ray μ-CT). Moreover, four fracture modes of the orthogonal PEEK and CF/PEEK printed composites are observed from large strain bending tests, and the mechanism of CFs affecting their flexural behavior is systematically studied.

## 2. Materials and Methods

### 2.1. Raw Materials and Filaments Preparation

PEEK (grade: ZYPEEK 550 G), supplied by Jilin Zhongyan High Performance Plastic Co., Ltd (Changchun, China), is an unreinforced grade for injection molding and extrusion. Chopped carbon fiber (grade: ZOLTEK MF150) with average fiber length of 100 μm, was purchased from ZOLTEK Co., Ltd (St. Louis, Missouri, USA). Prior to use the PEEK and carbon fiber (CF) were dried for at least 3 h at 150 °C. 

PEEK and CF/PEEK composite filaments were extruded by a Thermo Scientific^TM^ HAAKE^TM^ Process 11 Parallel twin-screw extruder (Thermo Fisher Scientific Co., Ltd, Karlsruhe, Germany) with 11 mm screw diameter, an L/D ratio of 40, and a single screw feeder. The twin-screw speed was set at 90 rpm, the single screw feeder speed was set at 5 rpm, and the processing temperature ranged from 365 to 385 °C. The amount of carbon fiber in CF/PEEK composites was about 5 wt %. The filaments for 3D printing were controlled with a diameter of 1.75 ± 0.05 mm.

### 2.2. Fabrication of 3D Printed Specimens

**Figure 1:**
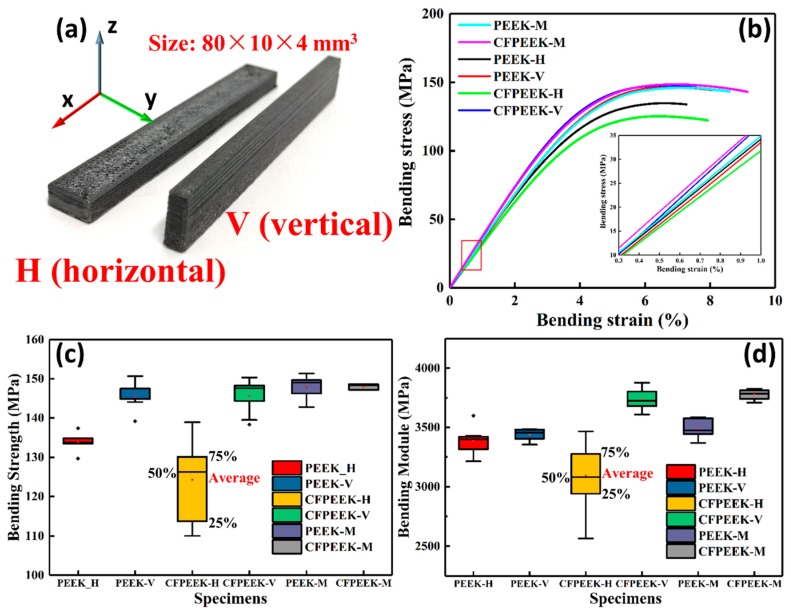
Flexural properties of printed and molding (M) polyether ether ketone (PEEK) and carbon fiber (CF)/PEEK specimens. (**a**) CF/PEEK bending specimens with orthogonal printing direction (horizontal and vertical) both printed according to ISO 178:2010 (size: 80 × 10 × 4 mm^3^). (**b**) Typical stress to strain curves of flexural experiments. The inset in the bottom right corner shows the linear range that determines the module of each bending specimen. The boxplots of (**c**) bending strength and (**d**) bending modulus of flexural specimens, which indicate the distribution of experimental data.

An open-source FUNMAT HT FDM 3D printer (INTAMSYS, Shanghai, China) was fed with the PEEK and CF/PEEK filaments to fabricate the specimens. The geometric models of flexural and short shear beam test specimens were designed by CATIA V5, according to the ISO 178:2003 [25] and ASTM D2344 [26]. The models’ files were imported to INTAM-SUITE, which is custom software for slicing and setting the printing parameters. All samples were printed with identical printing parameters, as shown in Table 1. In order to improve printing quality and reduce warping, three layers of polyvinyl pyrrolidone glue were successively applied onto the surface of a borosilicate glass building platform prior to the printing process. The flexural specimens in Figure 1a were fabricated into two orthogonal orientations (horizontal and vertical) to study the anisotropy of the flexural strength. The 3D printed specimens were tested without further thermal treatment.

### 2.3. Fabrication of Injection Molding Specimens

The prepared PEEK and CF/PEEK filaments both were pelleted and molded into flexural specimens (80 × 10 × 4 mm^3^, ISO 178:2010) by a Thermo Scientific^TM^ HAAKE^TM^ MiniJet injection molding system (Thermo Fisher Scientific Co., Ltd, Karlsruhe, Germany) under defined injection conditions. The injection temperature, injection pressure, injection duration, post-pressure, post-pressure duration, and mold temperature were set as 400 °C, 70 MPa, 10 s, 60 MPa, 10 s, and 180 °C, respectively. The pelleted filaments were dried for 5 h at 150 °C before use. After the injection molding process, the specimens were annealed for 2 h at 180 °C in an oven.

### 2.4. Flexural Testing of Fabricated Specimens

Flexural specimens were carried out on an Instron 5965 universal testing machine (INSTRON Co., Ltd, Norwood, MA, USA) with a 5 kN load capacity. Flexural tests (under three-point bending configuration) were carried out with a span length of 64 mm. The radius of two supports and central loading edge were 5.0 mm and the test speed was 2 mm/min according to ISO 178:2010. Prior to flexural testing, the length and width of a cross-section were averaged from the three times measuring of the midspan of each specimen using a Vernier caliper. At least five repeated specimens were tested, and data was averaged.

Short beam shear (SBS) stress tests were also carried out using the same testing machine with a three-point bending setup using a span of 24 mm (ASTM D2344). The horizontally printed specimens (40 × 12 × 6 mm^3^) were tested to investigate the interlayer adhesion of FDM parts at a speed of 1 mm/min. For each sample, the average value was obtained from at least five specimens. The stress of the SBS test was calculated from the equation [26]:

(1) $$S_{sbs}=0.75\;\frac{P_{max}}{bh}$$

where *S_sbs_* is short-beam shear stress (MPa). The *P_max_* is the maximum load recorded during the test, and *b*, *h* are the width and thickness of the specimen, respectively.

### 2.5. Thermal Properties Experiment (DSC)

Crystallinity analysis was carried out by a DSC 250 differential scanning calorimeter (TA Co., Ltd, New Castle, DE, USA) at a heating rate of 10 °C/min under nitrogen. Approximate 5.0 mg samples were put into an alumina crucible and heated from 50 to 400 °C. The samples were all cut from the fabricated flexural specimens in order to study the crystallinity of disparate positions of the specimens, such as shell, core, bottom, and top, respectively. All samples were tested without removing heat history. In addition, the crystallinity of molded specimens was set as the control. Crystallinity was determined by the equation [27]:

(2) $$\chi \left(\%\right)=\frac{\Delta H_{endo}+\Delta H_{exo}}{130}/v_{m}\times 100\%$$

where Δ*H_endo_*, Δ*H_exo_* are the integral area of endothermic peak and exothermic peak, respectively, *ν_m_* is the PEEK matrix content, and 130 J/g is the melting enthalpy of a 100% crystalline PEEK sample [28]. 

### 2.6. Morphological Observation (SEM and X-ray μ-CT)

The fracture surface of the 3D printed specimens was observed by a Quanta 650 FEG field-emission scanning electron microscope (Thermo Fisher Scientific Co., Ltd, Waltham, MA, USA) at an accelerating voltage of 10 kV. The observed samples were obtained from brittle fracture flexural specimens under liquid nitrogen. Before observation, the samples were coated with a gold conductive film for at least 60 s.

Imaging of 3D printed flexural specimens was performed on a Skyscan 1272 X-ray μ-CT system (Bruker Corp., Billerica, MA, USA). The X-ray source was 10 W with a voltage of 40 kV and a current of 250 mA. The X-ray detector was a 14 bit cooled CCD fiber-optically coupled to a scintillator. The image size of 4000 × 2672 pixels was selected. For printed specimens, the scanning resolution was around 5 mm/pixel and the image acquisition region was about 5 mm per specimen. For post-bending specimens, the scanning resolution was also around 5 mm/pixel and the image acquisition region was about 10.0 mm per specimen with a center of the midspan. After image acquisition, the 2D images were further processed to calculate porosity and pore distribution of specimens by Skyscan CTAn software. By means of combining with SEM and X-CT, the failure process and mechanism of printed parts could be investigated comprehensively.

## 3. Results and Discussion

### 3.1. Mechanical Behavior of Orthogonally Orientated Printed Composites

Flexural properties play an essential role in the mechanical performance of composites, and flexural experiments reproduce typical structure-loading characteristics, including tension, compression, and bending. Typical stress–strain curves of the printed and molded specimens, obtained by quasi-static three-point bending tests, are presented in Figure 1. All printed and molded samples exhibit an initial linear elastic deformation, and then reach a maximum of bending stress, but they do not break during the entire tests. These results show that the printed PEEK and CF/PEEK composites have similar high strength and toughness as the molded samples.

The specific values of the flexural strength and modulus are summarized in Table 2. The main flexural properties of the printed specimens were then compared to those of the molded neat PEEK samples in terms of the percentage of relative difference (% R.D.). For molded samples, the flexural strength of the CF/PEEK molded specimens were almost equal to neat PEEK. In comparison to the molded samples, the ultimate bending strength recorded for the printed composites declined by different amounts between the two orthogonal orientation printed composites. With the addition of CFs, the horizontally printed CF/PEEK composites exhibited a 7.1% lower value of than that of PEEK, which agrees with a previous report on the addition of carbon nanotubes (CNTs) [17]. Surprisingly, the vertically printed PEEK and CF/PEEK composites displayed a value of 146 MPa, which was similar to the value of the molded samples. Such a similar value between printed samples and molded samples is rare, but quite highly desired, for 3D printing. The effect of CF on the strength of the vertically printed parts will be explained in Section 3.2. Moreover, it is noteworthy that the bending strength of the horizontal CF/PEEK printed specimens were obviously lower than those of the other specimens, which suggests that the effect of CF on the PEEK composites needs to be systemically investigated.

It is well known that the flexural modulus can be used to evaluate the capacity of a sample’s resistance to bending deformation. A high flexural modulus means that specimens can bear larger bending stresses at the same strain. The data in Table 2 show that the incorporation of CF resulted in an 8.30% higher modulus for the CF/PEEK molded samples relative to those of the PEEK samples. A statistically significant increase in the modulus was also observed for the vertically printed samples. It is interesting to note that the highest (+7.16%) and lowest (−11.17%) values of the flexural modulus were obtained by the orthogonally printed CF/PEEK composites, which indicates the strong influence of building orientation on the modulus of the printed parts. The different results are possibly caused by unsatisfactory interlayer adhesion, or pores being induced by the addition of the CF. Further studies were then performed to investigate the mechanism of the variation of flexural behavior of the printed composites.

### 3.2. Multi-Scale Factors Affecting the Flexural Behavior of the Printed Composites

#### 3.2.1. Crystallization Behavior

The crystallinity of polymers and composites has a profound influence on the mechanical properties of printed samples, especially for PEEK-based printed parts, such as their elastic modulus, fracture toughness, yield strength, and thermal resistance [20]. Although the process parameters of the FDM (ambient temperature, printed bed temperature, printing speed) were consistent, the printed samples in different orientations undergo different thermal processes in the continuously changing thermal environment via the continuous introduction of the molten printing materials. Thus, it is important to investigate the crystallization behavior of the printed samples from different positions, such as the wall, core, bottom, and top.

**Figure 2:**
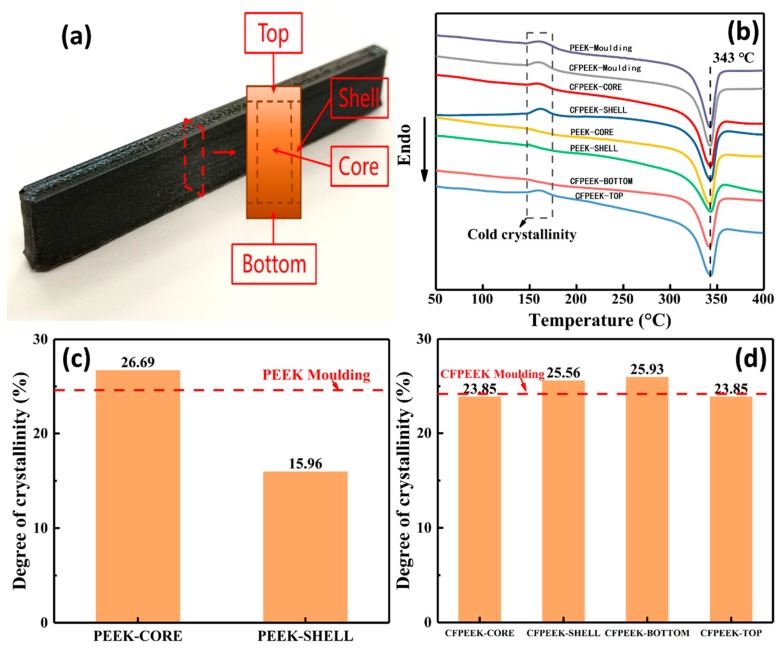
Differential scanning calorimetry (DSC) results of printed PEEK and CF/PEEK parts. (**a**) The position of DSC testing samples. (**b**) Heat flow curves of CF/PEEK and PEEK parts. (**c**) Otherness of crystallinity of shell and core of printed PEEK parts. (**d**) Corresponding crystallinity results of printed CF/PEEK parts.

DSC was used to investigate the crystallinity of the differently positioned printed samples. The results of the DSC and the crystallinity of samples are represented in Figure 2. Generally, two types of peaks can be observed in the heat flow curves—a cold crystallization peak and a melting peak. The former represents incomplete crystallization, and the latter indicates the polymer transforming from a high-elastic state to a molten state. As shown in Figure 2b, in addition to the bottom of the CF/PEEK samples and the core of the PEEK samples, imperfect crystallization was seen for each DSC sample. As for the molded samples, this phenomenon likely occurs due to the short molding time. However, the imperfect crystallization of the printed specimens may be due to the rapid heating and cooling processes, which were caused by a large temperature difference between the nozzle and the ambient air during extrusion [9]. Moreover, an interesting phenomenon of the different distributions of crystallinity between the PEEK and CF/PEEK printed specimens are recorded in Figure 2c–d. The crystallinity of the CF/PEEK printed parts appear to be more uniform compared to that of the PEEK parts. This phenomenon could possibly be caused by short CFs in the PEEK matrix acting as seeds that promote crystallization. Therefore, it was easier for a CF/PEEK printed part to obtain a homogeneous crystallinity compared to neat PEEK. In summary, the crystallization behavior of PEEK and CF/PEEK printed samples were highly dependent on the incorporation of CF and the processing condition.

#### 3.2.2. Layer-to-Layer Bonding Strength

Layer-to-layer bonding, which is an important parameter in 3D printing, greatly influences the mechanical properties of printed parts. As the most commonly used method to determine interlaminar shear adhesion of composite materials, a short beam shear (SBS) test was used to evaluate the layer-to-layer bonding of the printed parts, where the important data are summarized in Table 2. The printed PEEK samples exhibited a layer bonding value of 24.8 MPa, whereas the printed CF/PEEK samples presented a value of 19.1 MPa, which represents a significant decrease of 23.1%. The relative decrease in the layer bonding of the printed CF/PEEK samples could be explained by the addition of CFs into the PEEK matrix, which enhance the viscosity of the molten composites and change their surface characteristics (e.g., their surface roughness, surface tension, wettability, and adhesion) when in a molten state. These results agree with the lower flexural strength of the horizontally printed CF/PEEK specimens in comparison to that of the printed PEEK specimens.

#### 3.2.3. Microstructure

**Figure 3:**
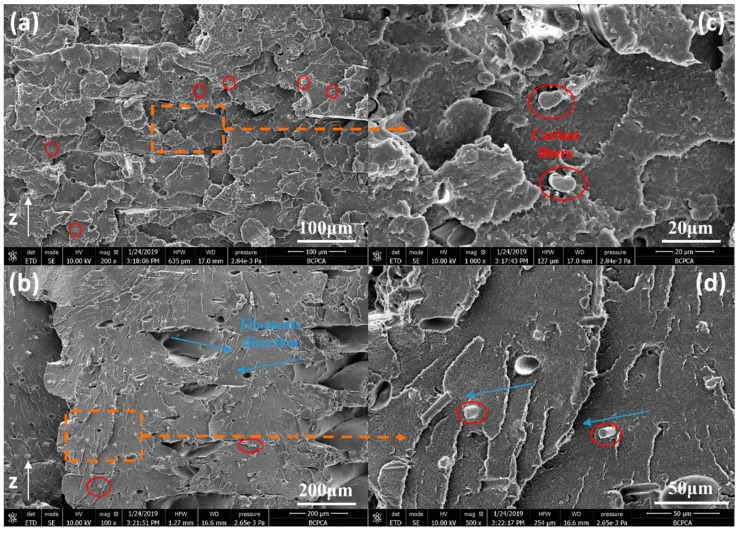
SEM of brittle flexural fractured cross-section of printed CF/PEEK parts. (**a**) The shell of printed CF/PEEK parts. (**b**) The core of printed CF/PEEK parts. (**c**), (**d**) The enlarged figures of (**a**), (**b**), respectively (red circles and blue arrows mean carbon fibers and direction of printing filaments, respectively).

The microstructure and porosity of the printed samples were examined by scanning electron microscope (SEM) and X-ray μ-CT. SEM micrographs from the fractured surfaces of the horizontally printed CF/PEEK samples are presented in Figure 3. The SEM micrographs reveal that the fractures on the surfaces of the printed CF/PEEK samples were composed of stacked layers, pores, and cracks, all of which correspond to the inherent layering approach of the 3D printing process. It can be concluded that the printed CF/PEEK samples are porous. For one thing, defects between layers and filaments can easily induce local stress concentrations, thus CF/PEEK composites are more likely to produce de-bonding fracture modes. For another, the cracks within filaments reduce the total area of material that can bear external forces. The reinforcing function of fibers and the additional pores introduced by the fibers largely counteract each other, which makes precisely predicting the macro-mechanics of CF/PEEK printed composites quite complex.

From Figure 3b–d, it can be observed that the CFs are well distributed in the PEEK matrix. Moreover, the majority of the CFs are aligned parallel to the printed direction. When the fibers and PEEK were simultaneously squeezed out from the heating head, shear stresses exist due to the different flow velocities of the melt along the radial direction. This energy allows the fibers to change their orientation relative to the printing direction. Such a reduction in the random spatial distribution of fiber orientation increases the anisotropy of the macro-mechanical properties of CF/PEEK printed parts. For semi-crystalline polymers, strong mechanical anisotropy of printed samples formed via the fused deposition process has been reported, such as for liquid crystal polymers, PEEK, etc. [2]. This feature of printed CF/PEEK composites harbors the potential to extend design methods by solving the unique requirements of mechanical boundary conditions.

#### 3.2.4. Porosity 

In order to further understand the mechanical behavior of the printed samples, it was essential to determine their porosity and pore distribution. In recent years, X-ray μ-CT has proven to be a useful tool for medical diagnoses and nondestructive ultrasonic detection of 3D printed samples [29]. The orthogonally printed flexural specimens before and after bending tests were scanned via X-ray μ-CT. From the results, we can see that the porosity of the two orthogonally printed CF/PEEK composites were larger than that of PEEK. This could be explained by the joint effect of higher viscosity in the molten state, higher thermal conductivity, a change in the contact angle characteristics, and the resin fiber interface. It is noteworthy that the porosities of the printed specimens before and after the bending tests exhibit great differences. Except for the horizontally printed PEEK composite, more pores appear in the structure of all samples, which can be attributed to structural damage incurred during the bending tests. As for the horizontally printed PEEK composite, the lower porosity after the bending test may suggest that the pores were compressed during the tests.

**Figure 4:**
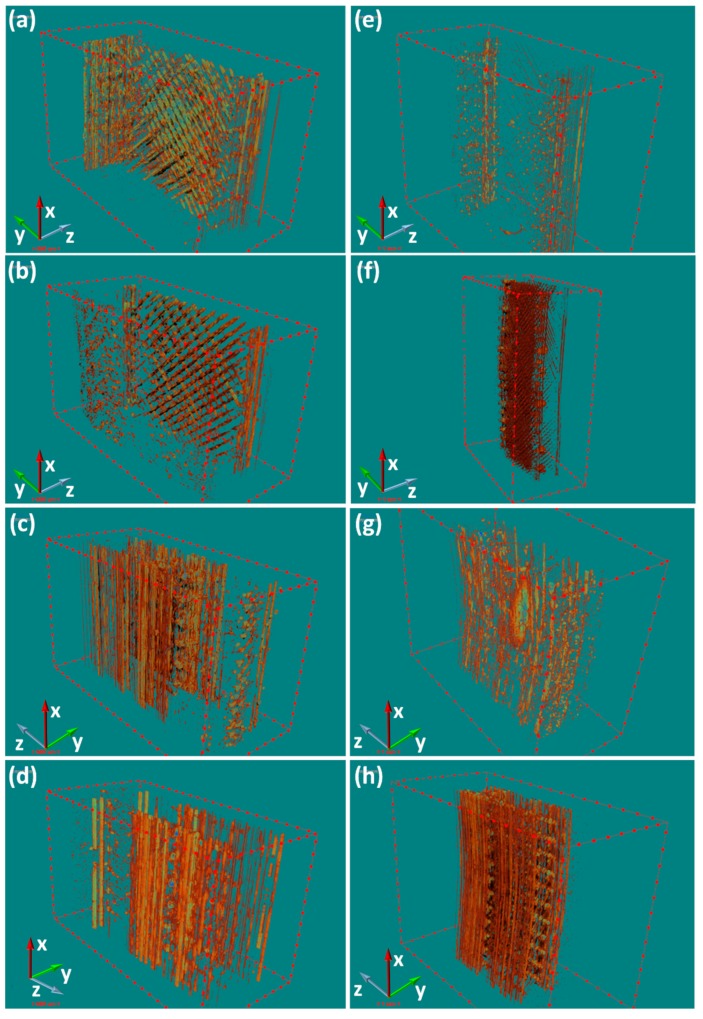
X-CT scanning of printed PEEK and CF/PEEK composites. Left (**a**–**d**): before bending; right (**e**–**h**): after bending. (**a**) (**b**) illustrate the horizontally printed parts and (**c**) (**d**) are the vertically printed parts. The Z-axis is building orientation, and the dark brown parts are pores. As for the horizontal and vertical parts, the external force is along the opposite direction of the Z-axis and the direction of the Y-axis, respectively.

An X-ray μ-CT scanned image and a 3D reconstructed pore distribution image of the horizontally printed PEEK and CF/PEEK samples are shown in Figure 4a,b, respectively. The frequency of pores and gaps was seen to increase with an increasing layer due to the variation of the temperature field used during the 3D printing. At least three types of pores and gaps can be observed in the 3D reconstructed pore distribution image. First, most of the gaps are mutually perpendicularly orientated, forming a layer-by-layer structure, in the inner core of the printed composites due to a lack of inter-line overlapping. Second, regular gaps exist between the inner core and shell owing to abrupt turns of the nozzle at the boundaries of the prefabricated shell. Third, irregularly distributed pores are present in the composites, which correspond to possible misruns and bubbles from the filaments.

As for the horizontally printed PEEK and CF/PEEK composites after the bending tests, the pores were squashed by compressive stresses, as shown in Figure 4e–f. Interestingly, the porosity of the printed PEEK and CF/PEEK composites show a variety of different behaviors. After the bending tests, the size and number of pores in the printed PEEK composites reduced, whereas those in the printed CF/PEEK composites increased and tended to gather around the side that underwent the applied tension. This phenomenon may be ascribed to the better bonding in the layers and filaments, and the relatively high toughness of PEEK (elongation at break: 40% strain rate). As for CF/PEEK, its reduced bonding and increased brittleness limited the amount of filament deformation and resulted in it being hard to cooperatively deform between filaments and layers, which will easily induce the cracks and pores. Moreover, on account of the lack of a strong shell for the horizontal specimens, the mutually perpendicular pores, which tended to concentrate on the tension-experiencing side, led to de-bonding between layers and filaments and a loss of carrying capacity. 

In comparison to the horizontally printed composites, gaps between the inner core and shell dominate the 3D reconstructed pore distribution of the vertically printed samples, as shown in Figure 4c,d. The gaps obviously enlarged with increasing distance to the heated plate. Meanwhile, the pores between adjacent layers were difficult to recognize. This could be related to the narrow cross section of each layer and the slightly higher surface temperature of the deposited layer, which could improve the interlayer strength and crystallinity of the composites. The above phenomena all demonstrate that the temperature of the composites during 3D printing plays an essential role in the quality of the printed parts.

More importantly, the change in the size and distribution of pores in the vertically printed composites after the bending tests showed enormous differences compared with the horizontally printed composites as recorded in Table 3. After the bending tests, the polarized gaps between the inner core and shell in the vertically printed composites have been transformed into uniform gaps, which illustrate the uniform deformation each layer experienced. However, an enormous crack can be seen in Figure 4g, which may have been caused by buckling. As the vertically printed samples were deposited perpendicular to the direction of force, all layers could stress, deform, and absorb major flexural energy uniformly, resulting in an enhanced maximum yield stress. These results agree with the high flexural strength of the vertically printed composites.

Even though the distribution of pores in the vertically printed PEEK composites changed, only a slight increase in porosity was found. Better layer-to-layer bonding could explain the small change in porosity for the PEEK samples. Meanwhile, it could be predicted that the difference in crystallinity between the inner core and the shell of the PEEK composite were responsible for the deformation. It is highly noteworthy that pores in both the inner core and shell of the vertically printed CF/PEEK composite after the bending tests can be distinguished in Figure 4h. The lower bonding strength caused by the addition of CFs into the PEEK could explain the increased porosity. The synergy of de-bonding in the inner core and the extension of gaps between the core and shell could dissipate part of the bending energy and assist in improving the flexural strength of the CF/PEEK samples.

### 3.3. Fracture Modes of the Printed Composites

**Figure 5:**
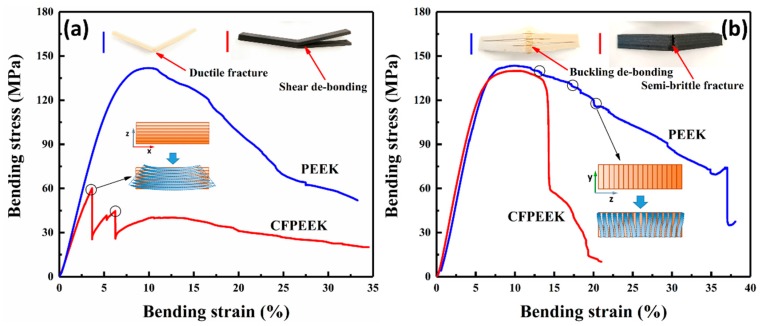
Bending fracture modes of (**a**) horizontally printed specimens, (**b**) vertically printed specimens.

Fracture modes, as a measure of the feasibility and reliability of printed composites, play an extraordinarily significant role in the practical application of composites. In order to observe any obvious fracture modes in the printed composites, a supplementary set of bending experiments, with a span of 32 mm and a loading edge (*R* = 2 mm), were performed to enlarge the degree of flexural strain. The stress–strain curves and the illustrations of fracture modes are shown in Figure 5. Four fracture modes can be observed from the typical stress–strain curves: (1) ductile fractures in the horizontally printed PEEK composites; (2) layer de-bonding of the horizontally printed CF/PEEK composites induced by shear deformation and dissatisfactory interlayer adhesion; (3) layer de-bonding of the vertically printed PEEK composites caused by buckling; and (4) typical semi-brittle fractures in the vertically printed CF/PEEK composites.

For the horizontally printed composites, the mismatch of deformation between each printed layer led to flexural properties that primarily depended on interlaminar adhesion. Moreover, the compressive stress was transformed to tensile stress in the neutral plane; thus, de-bonding was more likely to be generated at the special plane. Almost one-third of the horizontally printed CF/PEEK specimens experienced de-bonding fracture modes during bending, as shown in Figure 5a, whereas the horizontal PEEK specimens did not suffer de-bonding during bending. These different fracture phenomena were in agreement with the X-ray μ-CT, which confirms that the porosity of the CF/PEEK specimens was greater than the PEEK specimens, especially after bending. The addition of pores will further reduce the contact area between each layer and decrease the interlayer adhesion; hence, the bonding strength will easily arrive. The different fracture modes between the horizontally printed PEEK and CF/PEEK composites further demonstrate that interlayer bonding strength greatly limits the flexural properties of 3D printed composites.

**Figure 6:**
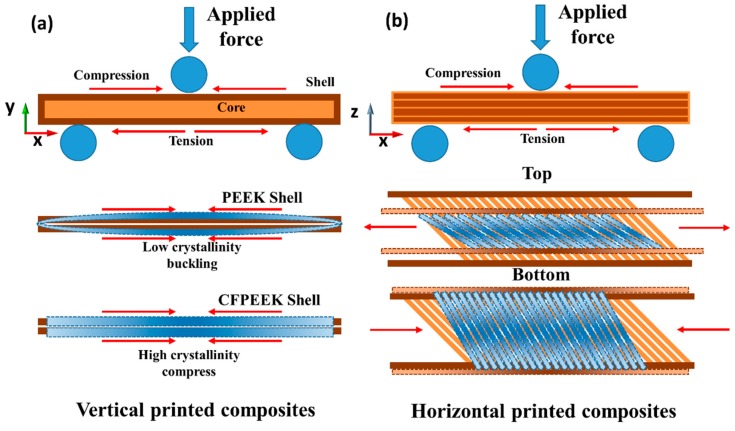
The major mode of deformation during bending. (**a**) The deformation mode of the vertically printed specimens. (**b**) The deformation mode of horizontally printed specimens.

Compared with the horizontal specimens, the fracture modes of the vertically printed PEEK and CF/PEEK composites were obviously different, as shown in Figure 5b, which can be explained by the DSC results. A high crystallinity shell has a higher stiffness than a low crystallinity shell, which means that the shell of the CF/PEEK specimens makes it harder to generate buckling at the same compressive stress shown in Figure 6a. On the contrary, the toughness of a high crystallinity shell is less than a low crystallinity shell; thus, in accordance with the stress-shielding effect, the shells of the CF/PEEK printed specimens will bear more stress than that of PEEK samples. Moreover, the neutral plane was moving down with the increasing degree of buckling, which induces the bending stress on the bottom of printed PEEK specimens that can be relatively reduced. As a result, the shells of the CF/PEEK printed specimens break at 15% of the flexural strain, while the value of the PEEK printed specimens was almost 40%. Although the interlayer adhesion of PEEK was better, it was still hard for the layers to withstand the perpendicular force. Thus in-plane buckling, which was induced by the low crystallinity shells of the PEEK samples, can easily destroy the well-bonded layers. Under the influence of the above factors, the fracture mode of the CF/PEEK vertical specimens was predominantly brittle fracture, while in-plane buckling dominated for the PEEK vertical specimens.

### 3.4. Mechanism of CFs Affecting Flexural Behavior of Orthogonal Printed Specimens

Short CFs, as a reinforcement phase, impact printed CF/PEEK composites, which can affect their porosity, stiffness, anisotropy, and interlayer bonding, all of which conjointly influence their ultimate flexural properties. However, the degree that each factor affects the horizontally and vertically printed composites is different. The mechanism of CFs influencing the flexural behavior needs further analyses from microstructure.

**Figure 7:**
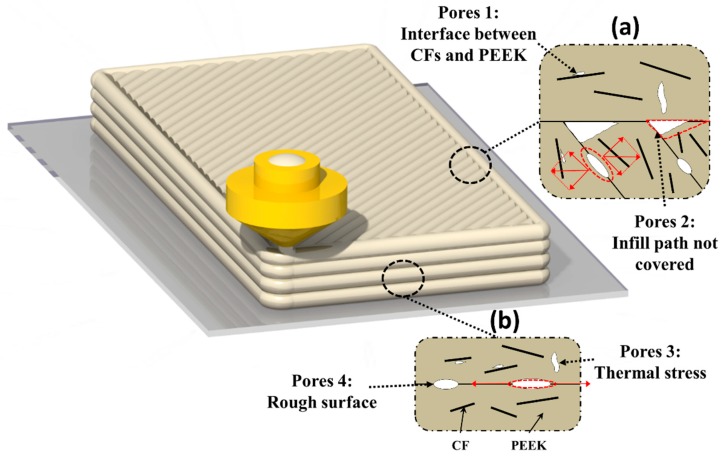
The effect of pores induced by CFs on the flexural behavior of orthogonal printed specimens. The main microstructure of (**a**) horizontal printed parts and (**b**) vertical printed parts that bear the bending stress.

It is well known that the tensile stress induced by bending plays a critical role in destroying structure, thus understanding the porous microstructure which bears tensile stress is quite significant for investigating the flexural behavior of orthogonal printed CF/PEEK composites. As shown in Figure 7, there exist four types of pores distributed in printed specimens, which are similar to those of [19]. Except for the type 2 pores, the CFs will enlarge the amounts of other pores due to the increasing viscosity and thermal conductivity (as demonstrated by the X-ray μ-CT results). Among these pores, the type 4 and type 2 cause the decreasing bonding strength of filaments and layers, and type 3 and type 1 reduce the area of material to resist tensile stress, which may decrease the break strength of printed specimens. In the shell of a vertically printed specimen, most pores were distributed along the direction of stress (except for the type 3 pores). For the horizontal specimens, however, most pores were aligned perpendicular to the component force of the tension, especially the type 2 pores, which more easily enlarge under tension and lead to the infill losing the capacity to bear stress. Moreover, the CFs enhance the stiffness and anisotropy of each printed filament, which makes the shell of the vertical parts more able to resist directional stresses. The vertical printed CF/PEEK composites avoid the major negative effects and utilize the positive effects of CFs. As a result, the flexural performance of vertical printed composites is better than that of horizontal composites and the vertical printed CF/PEEK can possess the similar flexural properties with molded specimens. In general, the difference of flexural behavior indicates that correct design methods and printing processes should make best use of the advantages, and try to bypass the disadvantages, of short CFs to reinforce composite 3D printed parts via FDM to obtain satisfactory mechanical properties.

## 4. Conclusions

PEEK and CF/PEEK composites with orthogonal orientations were successfully printed via FDM. It was found that the flexural strength and module of vertical printed CF/PEEK composites increased 17.7% and 20.6%, respectively, compared to horizontal specimens. Compared with the neat PEEK printed parts, the addition of SCFs raised the uniform nucleation process and porosity of PEEK during FDM. X-ray μ-CT experiments revealed that the porosity of horizontal, vertical printed PEEK, and CF/PEEK composites was enlarged by −72.3%, +14.5%, +168.7%, and +87.7% after bending, respectively. Four fracture modes (semi-brittle fractures, ductile fractures, buckling, and interlayer de-bonding) of printed composites were observed in our bending tests with large strain. In addition, the influence of CFs on flexural behavior of orthogonal printed specimens was investigated. The design of a printing route along the stress orientation that cooperates with the incorporation of a reinforced phase into the matrix provides an effective method to enhance the mechanical properties of composites and enlarges the application of 3D printing in lightweight design fields. This study will be helpful to designers to investigate the influence of microstructures on printed composites during the printing process.

## Figures and Tables

**Table 1:** Parameters of fused deposition modeling (FDM) processing.

Items	Parameters
Diameter of nozzle	0.4 mm
Nozzle temperature	400 °C
Ambient temperature	90 °C
Heat platform temperature	160 °C
Nozzle moving speed	15 mm/s
Layer thickness	0.1 mm
Raster angle	+45°/−45°
Air gap	0.18 mm

**Table 2:** Main bending properties of PEEK and CF/PEEK printed samples.

Samples	σ_max_ (MPa)	%R.D. *^a^*	E (GPa)	%R.D.	SBS (MPa)	%R.D.
PEEK-M	148 ± 3.4	—	3.49 ± 0.09	—	—	—
CF/PEEK-M	148 ± 0.8	—	3.78 ± 0.05	+8.31	—	—
PEEK-H	134 ± 2.8	−9.46	3.39 ± 0.11	−2.87	24.8 ± 0.7	—
PEEK-V	146 ± 3.3	−1.35	3.44 ± 0.05	−1.43	—	—
CF/PEEK-H	124 ± 9.6	−16.2	3.10 ± 0.27	−11.2	19.1 ± 3.2	−23.1
CF/PEEK-V	146 ± 4.2	−1.35	3.74 ± 0.09	+7.16	—	—

*^a^* percent relative difference. SBS: short beam shear.

**Table 3:** The result of porosity analysis via X-ray computed tomography (X-CT).

Samples	Porosity (%) before Bending	Porosity (%) after Bending	Δ(%)
PEEK-H	2.60	0.72	−72.3
PEEK-V	2.62	3.00	+14.5
CF/PEEK-H	3.00	8.06	+168.7
CF/PEEK-V	4.38	8.22	+87.7
